# Metastatic Lymphadenopathy As the Initial Presentation of Gastric Carcinoma: A Case Report

**DOI:** 10.7759/cureus.117226

**Published:** 2026-09-30

**Authors:** Nawraa H Mohamed, Ahmed Alsharakhat, Fatema Mohamed

**Affiliations:** 1 Internal Medicine, Salmaniya Medical Complex, Manama, BHR; 2 Pathology and Laboratory Medicine, Salmaniya Medical Complex, Manama, BHR; 3 Gastroenterology and Hepatology, Salmaniya Medical Complex, Manama, BHR

**Keywords:** case report, gastric cancer, metastatic lymphadenopathy, signet ring cell carcinoma, young onset gastric cancer

## Abstract

Gastric cancer in younger adults is relatively uncommon and may present with nonspecific or atypical clinical manifestations, which can contribute to delayed recognition. Signet-ring cell adenocarcinoma is a distinct histological subtype of gastric cancer that has been described in association with diffuse growth patterns and advanced disease in some patients. These features may contribute to diagnostic challenges, particularly when gastrointestinal symptoms are absent or nonspecific.

We report the case of a 41-year-old male who was referred for evaluation of incidentally discovered abnormal liver function tests with an obstructive pattern during routine assessment. The patient reported approximately 10 kg of unintentional weight loss over six months but denied any localized gastrointestinal symptoms.

Initial abdominal ultrasound revealed only mild fatty liver changes. Subsequent evaluation with magnetic resonance cholangiopancreatography (MRCP) demonstrated multiple enlarged conglomerate lymph nodes involving the porta hepatis, superior mesenteric, and peripancreatic regions. Histopathological assessment of a mesenteric lymph node biopsy revealed metastatic carcinoma of probable gastrointestinal origin, with tumor cells positive for AE1/AE3, CK20, CK19, and CDX2 on immunohistochemistry. Esophagogastroduodenoscopy (EGD) identified a large ulcerative gastric lesion along the greater curvature of the stomach. Histopathological analysis of gastric biopsies demonstrated poorly differentiated signet-ring cell adenocarcinoma associated with *Helicobacter pylori* infection. The patient was referred to the oncology service for further management.

Younger patients with gastric cancer may present with advanced disease and atypical manifestations, contributing to delayed diagnosis. This case is notable for its atypical presentation with obstructive liver function abnormalities and metastatic intra-abdominal lymphadenopathy preceding identification of the primary gastric lesion. The case emphasizes the diagnostic importance of histopathological and immunohistochemical evaluation in patients with unexplained intra-abdominal lymphadenopathy.

This case highlights the importance of maintaining a broad differential diagnosis in patients presenting with unexplained laboratory abnormalities or atypical radiological findings, even in the absence of gastrointestinal symptoms. Prompt histopathological evaluation, appropriate immunohistochemical analysis, and timely endoscopic assessment can facilitate identification of the primary tumor and establishment of the diagnosis.

## Introduction

Gastric cancer remains one of the leading causes of cancer-related morbidity and mortality worldwide, despite a gradual decline in incidence in several regions. According to Global Cancer Observatory (GLOBOCAN) 2020, gastric cancer was estimated to be the fifth most commonly diagnosed malignancy and the fourth leading cause of cancer-related death globally [1]. Although the disease predominantly affects older individuals, gastric cancer in younger adults is increasingly recognized and may exhibit distinct clinicopathological characteristics compared with the disease in older patients [2,3].

Young-onset gastric cancer has been associated with a higher frequency of poorly differentiated histology and distinct clinicopathological features compared with gastric cancer in older patients [3,4]. Diagnosis may be delayed because symptoms can be nonspecific, and the possibility of malignancy may be less readily considered in younger individuals [4]. Younger patients may also present with lymph node involvement or advanced disease, although reported outcomes vary across studies [2,3].

We report a case of poorly differentiated gastric signet-ring cell adenocarcinoma in a 41-year-old male who presented with significant unintentional weight loss in the absence of localized gastrointestinal symptoms. Liver function abnormalities with an obstructive pattern were incidentally detected during routine assessment, and subsequent imaging revealed extensive intra-abdominal lymphadenopathy. This case highlights the diagnostic challenges associated with atypical presentations of gastric cancer in younger adults and emphasizes the importance of maintaining a high clinical suspicion for occult malignancy in patients with unexplained laboratory or radiological abnormalities.

## Case presentation

Patient history

A 41-year-old male with no significant past medical history was referred to the gastroenterology clinic for evaluation of deranged liver function tests identified during a routine check-up at a primary healthcare center. The patient reported approximately 10 kg of unintentional weight loss over the preceding six months; however, he denied abdominal pain, nausea, vomiting, pruritus, or fever. He reported no history of alcohol consumption, smoking, or illicit drug use. Additionally, he had no prior history of blood transfusions. There was no significant family history of malignancy or other serious medical conditions. The patient had no previous diagnosis of or treatment for *H. pylori *infection.

Physical examination

On physical examination, the patient appeared well and in no apparent distress. His vital signs were within normal limits. Abdominal examination revealed a soft, non-tender abdomen with no evidence of organomegaly or palpable masses. The remainder of the physical examination was unremarkable.

Laboratory investigations

Initial laboratory investigations revealed deranged liver function tests demonstrating an obstructive pattern (Table 1). Tumor marker evaluation showed an elevated carbohydrate antigen 19-9 (CA 19-9) level of 134 U/mL (reference ≤37 U/mL) and a mildly elevated carcinoembryonic antigen (CEA) level of 7.2 µg/L (reference ≤2.5 µg/L in non-smokers). Alpha-fetoprotein (AFP) was within normal limits at 4.8 µg/L (reference range: 0-8 µg/L). Viral and autoimmune serologies were negative. In addition, the CBC, including WBC count, hemoglobin level, and platelet count, as well as the coagulation profile, was within normal limits.

**Table 1:** Laboratory results of the patient. INR: International normalized ratio; CA 19-9: Carbohydrate antigen 19-9; CEA: Carcinoembryonic antigen; AFP: Alpha-fetoprotein.

Parameter	Result	Reference range	Units
WBCs	5.56	3.4-9.6	×10⁹/L
Hemoglobin	13.3	13.2-16.6	g/dL
Platelets	261	150-400	×10⁹/L
INR	1.1	0.61-1.17	-
Prothrombin time	12.5	10.7-13.9	s
Albumin	46	35-52	g/L
Total bilirubin	22	5-21	µmol/L
Direct bilirubin	6	≤5	µmol/L
Indirect bilirubin	15.9	≤18	µmol/L
Alkaline phosphatase	154	45-116	U/L
Alanine aminotransferase	85	10-49	U/L
Gamma-glutamyl transferase	306	≤73	U/L
Anti-liver/kidney antibody	Negative	-	-
Anti-smooth muscle antibody	Negative	-	-
Anti-mitochondrial antibody	Negative	-	-
CA 19-9	134	≤37	U/mL
CEA	7.2	≤2.5	µg/L
AFP	4.8	0-8	µg/L

Imaging studies

An initial abdominal ultrasound was performed and was unremarkable except for mild fatty liver. Subsequent CT of the abdomen demonstrated multiple enlarged intra-abdominal lymph nodes involving the porta hepatis, peripancreatic, and superior mesenteric regions (Figure 1).

**Figure 1:**
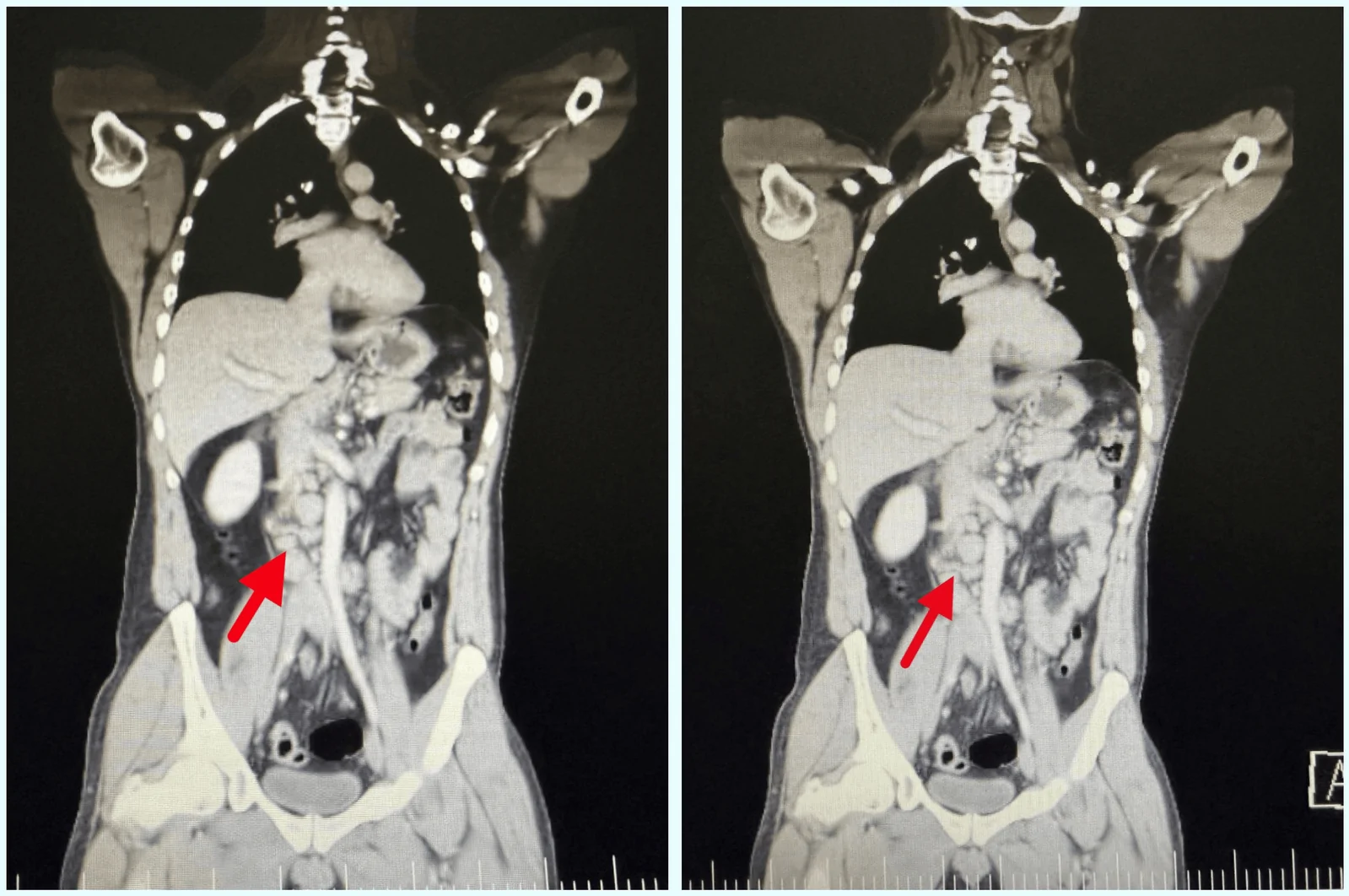
Abdominal CT scan demonstrating multiple enlarged intra-abdominal lymph nodes (arrows).

Further evaluation with magnetic resonance cholangiopancreatography (MRCP) showed multiple enlarged, conglomerate lymph nodes in the porta hepatis, superior mesenteric, and peripancreatic regions. The largest lymph node at the hepatic hilum measured 2.4 cm, while the largest peripancreatic and superior mesenteric lymph nodes measured 1.7 cm and 1.8 cm, respectively. The lymph nodes demonstrated intermediate signal intensity on T1-weighted images, mild hyperintensity on T2-weighted images, and restricted diffusion. The common bile duct (CBD) measured approximately 3 mm in diameter, with indentation by the porta hepatis lymph nodes and associated prominence of the intrahepatic biliary ducts (Figure 2).

**Figure 2:**
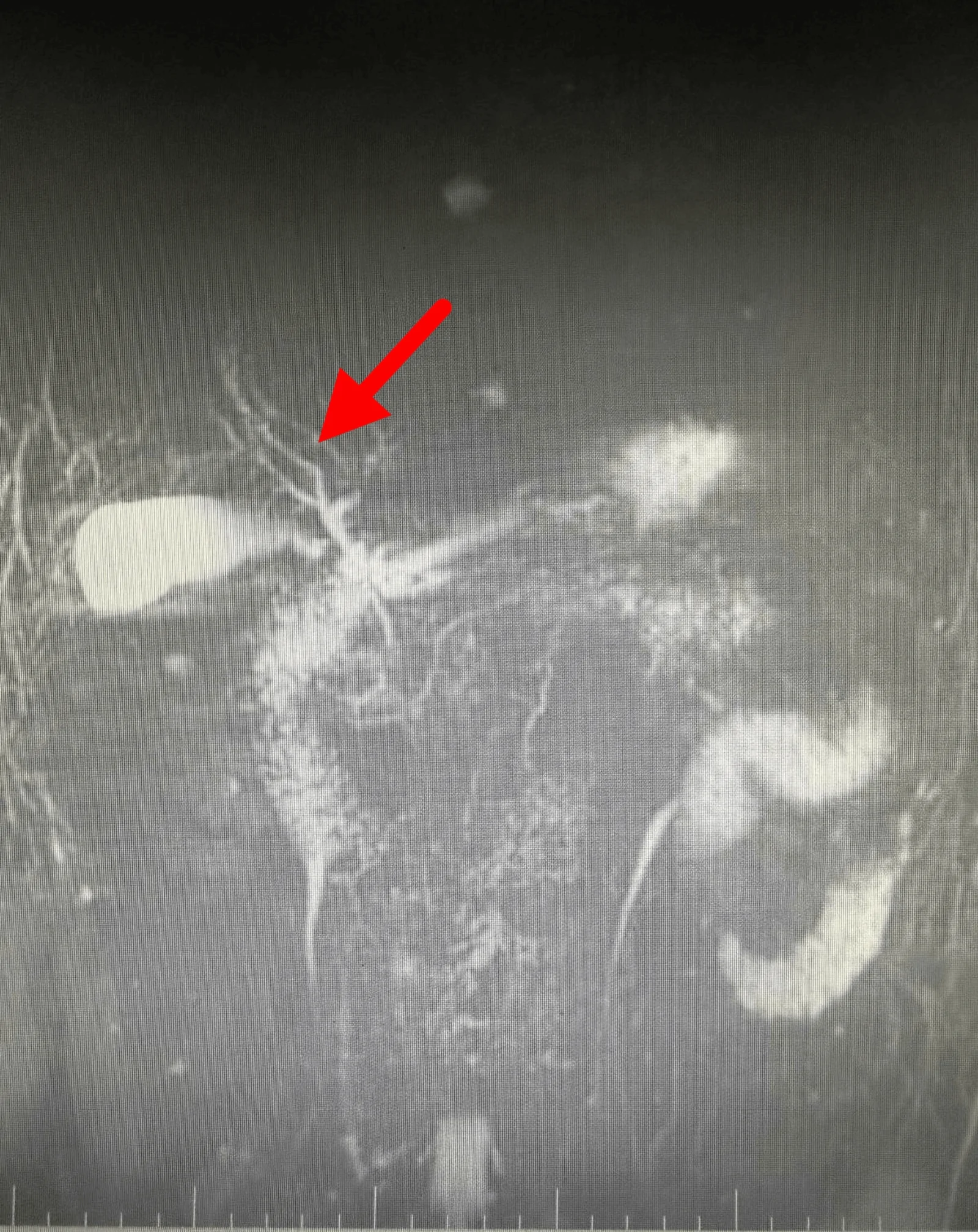
Magnetic resonance cholangiopancreatography (MRCP) demonstrating prominence of the intrahepatic bile ducts (arrow).

Subsequently, the patient underwent a mesenteric lymph node biopsy for histopathological evaluation.

Endoscopic examination and biopsy

Histopathological examination of the excised lymph node demonstrated infiltration by malignant epithelial cells arranged in clusters and dispersed singly. The tumor cells exhibited marked pleomorphism, round hyperchromatic nuclei, and clear cytoplasm. Immunohistochemical studies showed positivity for cytokeratin AE1/AE3, CK20, CK19, and CDX2, while the tumor cells were negative for Hep-Par1, OCT4, RCC, CD20, and CD30. The overall findings were consistent with metastatic carcinoma, favoring a gastrointestinal primary origin (Figure 3).

**Figure 3:**
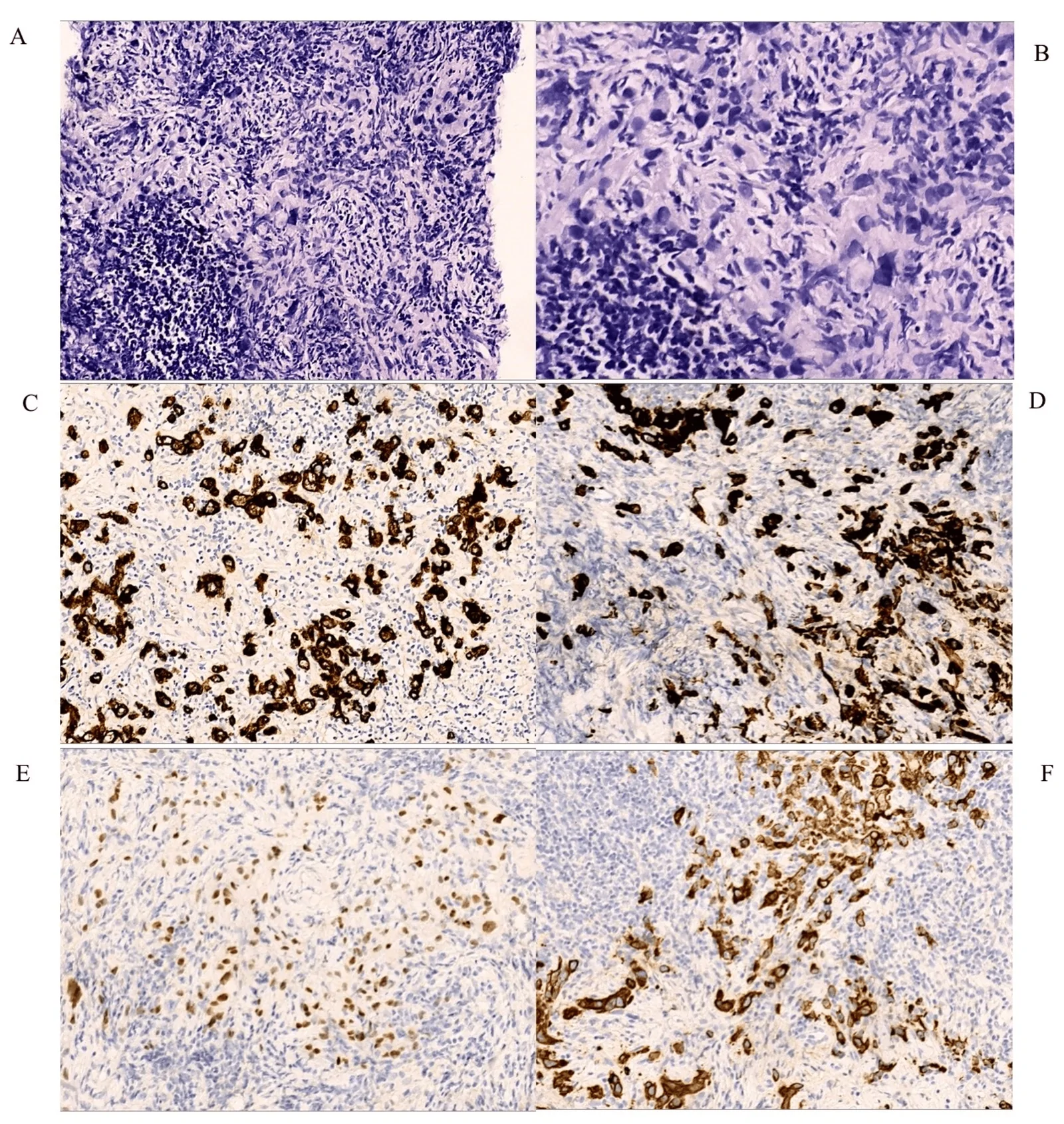
Histopathological and immunohistochemical findings of the excised lymph node. (A) Lymph node infiltrated by malignant epithelial cells within lymphoid tissue (H&E, ×20). (B) Tumor cells arranged in clusters and dispersed singly, showing pleomorphic hyperchromatic nuclei and clear cytoplasm (H&E, ×40). Immunohistochemical staining demonstrating tumor cell positivity for (C) AE1/AE3, (D) CK20, (E) CDX2, and (F) CK19 (all ×20). CK: Cytokeratin; CDX2: Caudal-type homeobox 2.

Further evaluation with esophagogastroduodenoscopy (EGD) revealed a large, suspicious, clean-based ulcer with raised edges located along the anterior wall of the greater curvature of the stomach, with edematous margins. Multiple biopsies were obtained from the lesion.

Microscopic examination of the gastric biopsies demonstrated poorly differentiated malignant cells infiltrating the lamina propria, including cells with signet-ring morphology. The surrounding lamina propria showed prominent lymphoid aggregates with follicular formation, and *H. pylori* organisms were identified. Immunohistochemical staining showed positivity for AE1/AE3 in the neoplastic cells, while leukocyte common antigen (LCA) highlighted the accompanying lymphoid infiltrate. The lesion was diagnosed as poorly differentiated adenocarcinoma with signet-ring cell morphology; definitive tumor classification was deferred pending examination of the resection specimen (Figure 4).

**Figure 4:**
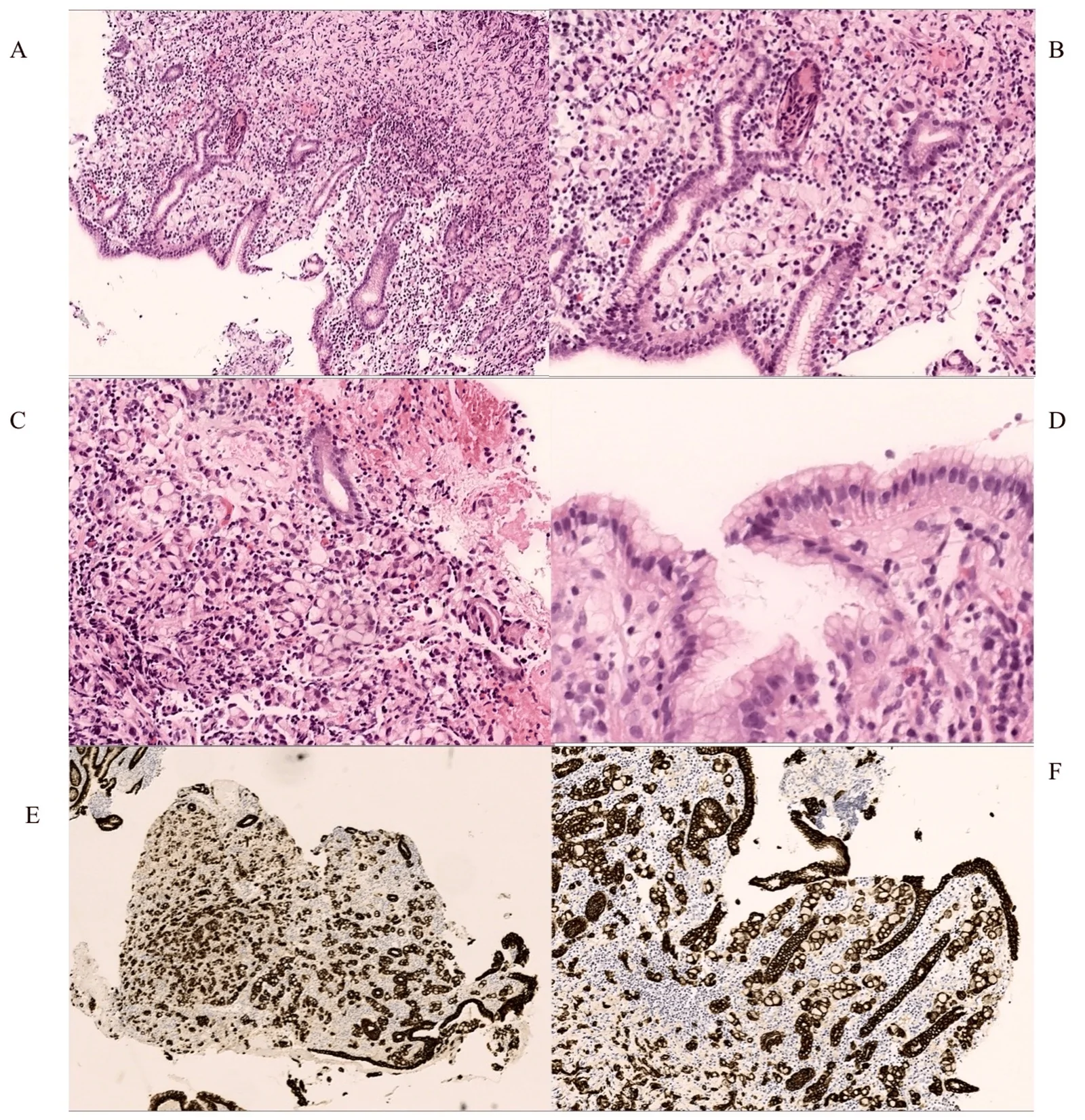
Histopathological and immunohistochemical findings of the gastric biopsy. (A) Poorly differentiated malignant cells infiltrating the lamina propria (H&E, ×10). (B, C) Higher-power views showing tumor cells with signet-ring cell morphology (H&E, ×20). (D) Presence of *Helicobacter pylori* organisms (H&E, ×40). Immunohistochemical staining demonstrating AE1/AE3 positivity in the tumor cells at (E) low magnification (×5) and (F) higher magnification (×20).

Staging and treatment

Further staging with CT of the chest showed no evidence of distant organ metastases. The case was subsequently referred to the multidisciplinary tumor board for discussion and treatment planning. The patient was then referred to the oncology service for further management and was commenced on chemotherapy.

## Discussion

Gastric cancer remains a major global health burden and is the fifth most commonly diagnosed malignancy and the fourth leading cause of cancer-related mortality worldwide. According to GLOBOCAN 2020 estimates from the International Agency for Research on Cancer, more than one million new gastric cancer cases and approximately 769,000 related deaths occur annually worldwide [1]. However, gastric cancer occurring in younger individuals remains relatively uncommon and often presents with unique clinicopathological characteristics compared with older populations [1].

Several studies have demonstrated that gastric cancer in younger patients tends to exhibit more aggressive biological behavior, advanced stage at diagnosis, and poorer histopathological differentiation. Al-Refaie WB et al. reported that younger patients frequently present with diffuse-type adenocarcinoma and signet-ring cell histology, despite often having fewer traditional risk factors associated with gastric malignancy [2]. Similarly, Takatsu Y et al. observed that diffuse infiltrative tumors and poorly differentiated histology were significantly more common among younger patients compared with older cohorts [3]. In the present case, the patient demonstrated poorly differentiated signet-ring cell gastric adenocarcinoma, consistent with findings reported in the literature regarding younger-onset gastric cancer.

Although published reports have included patients across a range of ages, with varying age thresholds used to define young-onset gastric cancer, our patient was 41 years old, which still represents a relatively young age for gastric carcinoma presentation. Studies by López-Basave HN et al. and Dhobi MA et al. suggested that younger patients may experience delayed diagnosis due to lower clinical suspicion of malignancy in this age group and the nonspecific nature of early symptoms [4,5]. Interestingly, unlike several reported cases, our patient had no localized gastrointestinal symptoms and presented with incidentally discovered obstructive liver function test abnormalities during routine evaluation. This atypical presentation emphasizes the diagnostic challenge associated with gastric cancer in younger individuals and highlights the importance of maintaining a broad differential diagnosis even in patients without gastrointestinal symptoms.

In the present case, initial abdominal ultrasound demonstrated only mild fatty liver changes, while MRCP revealed extensive lymphadenopathy involving the porta hepatis, superior mesenteric, and peripancreatic regions. Metastatic lymphadenopathy as the initial manifestation of gastric cancer has been described in several case reports involving young adults. Torere BE et al. described a 23-year-old female with metastatic gastric signet-ring cell carcinoma initially presenting with malignant pleural effusion [6], while Bendimya M et al. reported an unusual presentation with breast metastasis in a young patient [7]. These reports emphasize the aggressive metastatic potential of signet-ring cell gastric carcinoma and its tendency to present with extragastric manifestations before identification of the primary lesion. Similarly, in our patient, the diagnosis was initially suspected following histopathological examination of a mesenteric lymph node biopsy, which demonstrated metastatic carcinoma with immunohistochemical features suggestive of gastrointestinal origin.

Immunohistochemistry played an important role in the initial characterization of the metastatic carcinoma in this case. Positivity for CK20, CK19, CDX2, and AE1/AE3 supported a gastrointestinal epithelial origin and guided further endoscopic evaluation. Subsequent EGD identified an ulcerative gastric lesion, with biopsy confirming poorly differentiated signet-ring cell adenocarcinoma. Signet-ring cell carcinoma is a histological type that may be associated with diffuse infiltration and advanced disease. Reports by Alhalabi MM et al., Daboul B et al., and Wang H and Li C have described gastric cancer in younger patients, including cases with diffuse or poorly differentiated histology and advanced disease at presentation [8-10].

Another notable finding in our patient was the presence of associated *H. pylori* infection identified on histopathological examination. *H. pylori* infection has been associated with gastric adenocarcinoma and may contribute to gastric carcinogenesis, including among younger patients [8,10,11]. However, its specific relationship with diffuse-type and signet-ring cell carcinoma remains less clearly defined. In the present case, the coexistence of *H. pylori* infection and signet-ring cell adenocarcinoma was observed; however, a direct causal relationship between *H. pylori* infection and signet-ring cell histology cannot be established.

Young-onset gastric cancer has been associated with poorer prognosis in multiple studies, largely attributed to advanced disease stage at diagnosis and aggressive histopathological features rather than age alone. López-Basave HN et al. questioned whether poorer outcomes in young patients reflect inherently aggressive tumor biology or delays in diagnosis, concluding that advanced presentation significantly contributes to adverse outcomes [4]. Likewise, Mangaza A et al. described three young adult patients presenting with advanced-stage disease and emphasized the importance of early recognition and timely endoscopic evaluation [11].

The present case further illustrates the importance of considering occult gastrointestinal malignancy in patients presenting with unexplained intra-abdominal lymphadenopathy or cholestatic liver enzyme abnormalities, even in the absence of gastrointestinal symptoms. Early tissue diagnosis through lymph node biopsy and the use of immunohistochemical markers were essential in guiding further diagnostic evaluation in this patient.

In conclusion, this case highlights the importance of considering occult gastric malignancy in younger patients with unexplained lymphadenopathy or abnormal liver function tests, even in the absence of classical gastrointestinal symptoms. Tissue diagnosis, immunohistochemical evaluation, and subsequent endoscopic assessment can facilitate identification of the primary tumor and establishment of the diagnosis.

Highlights

This case demonstrates that gastric signet-ring cell carcinoma may present without localized gastrointestinal symptoms and that obstructive liver function abnormalities may represent an initial clue to occult malignancy. Intra-abdominal lymphadenopathy may precede identification of the primary gastric lesion, and immunohistochemistry can support the suspected gastrointestinal origin of metastatic carcinoma. Early endoscopic evaluation should be considered in patients with unexplained metastatic lymphadenopathy.

## Conclusions

This case highlights the importance of maintaining a broad differential diagnosis in patients presenting with unexplained laboratory abnormalities or nonspecific clinical findings, even in the absence of localized gastrointestinal symptoms. Young-onset gastric cancer, including signet-ring cell adenocarcinoma, may present with atypical features that can make diagnosis challenging. Prompt histopathological evaluation, appropriate immunohistochemical analysis, and timely endoscopic assessment can facilitate identification of the primary tumor and establishment of the diagnosis.
